# Salvage robotic-assisted radical prostatectomy after targeted high-intensity focused ultrasound: a single-center study on feasibility, oncological and functional outcomes

**DOI:** 10.3389/fonc.2026.1711853

**Published:** 2026-02-06

**Authors:** Ludovica Cella, Vittorio Fasulo, Stefano Moretto, Edoardo Beatrici, Andrea Piccolini, Pier Paolo Avolio, Davide Maffei, Roberto Contieri, Alessandro Tallari, Valerio Mallia, Alessandro Uleri, Alberto Saita, Rodolfo Hurle, Paolo Casale, Massimo Lazzeri, Marco Paciotti, Nicolò Maria Buffi, Giovanni Lughezzani

**Affiliations:** 1 Department of Biomedical Sciences, Humanitas University, Milan, Italy; 2 Department of Urology, Scientific Institute for Research, Hospitalization and Healthcare (IRCCS) Humanitas Research Hospital, Milan, Italy; 3 Urology Service, Assistance Publique – Hôpitaux de Paris (AP-HP), Hopital Tenon, Assistance-Publique Hopitaux de Paris, Sorbonne Universite, Paris, France; 4 Uro-Gynecological Department, Istituto Nazionale Tumori di Napoli, Scientific Institute for Research, Hospitalization and Healthcare (IRCCS) “G. Pascale”, Naples, Italy; 5 Department of Urology, Josep Trueta Hospital, Girona, Spain

**Keywords:** focal therapy failure, HIFU, HIFU failure, high-intensity focused ultrasound, high-intensity focused ultrasound failure, salvage RARP, salvage robotic-assisted radical prostatectomy, targeted focal therapy

## Abstract

**Background/Objectives:**

Targeted High-Intensity Focused Ultrasound (HIFU) is an emerging treatment option for localized prostate cancer (PCa), aiming to balance oncological control with functional preservation. However, data on the feasibility and outcomes of salvage robotic-assisted radical prostatectomy (sRARP) after targeted HIFU remain limited. This study aimed to evaluate feasibility, functional and early oncological outcomes of sRARP in this specific setting.

**Methods:**

Among 112 men treated with targeted HIFU for localized prostate cancer, 15 underwent sRARP between June 2022 and June 2025. The primary outcome was surgical feasibility and safety, assessed by conversion rate, operative time, nerve-sparing approach, lymphadenectomy, positive surgical margins (PSM) at frozen section and final pathology, and intraoperative complications. Secondary outcomes included urinary continence (full or social continence at 1 year), erectile function (with or without phosphodiesterase type 5 inhibitors at 1 year), PSA levels at 45 days and one year after surgery, and postoperative complications.

**Results:**

All procedures were completed robotically without intraoperative complications or conversions. Bilateral nerve-sparing was performed in 13 patients (87%). PSM were found in three (20%) patients at frozen section and in two patients (13%) at final pathology; one patient (11%) had lymph node metastases. Early complications occurred in three patients (20%), with only one Clavien Dindo grade ≥3 event. At 1-year follow-up, one patient (8%) had PSA >0.2 ng/mL; nine patients (75%) were continent and six (50%) reported preserved erectile function. The median follow-up was 14 months (IQR 10.8–16.5; 95% CI).

**Conclusions:**

sRARP after targeted HIFU is feasible and safe, with favorable early oncological and functional outcomes. These findings suggest that targeted HIFU may be a viable option in selected patients, without compromising future curative strategies in case of recurrence.

## 1 Introduction

Robot-assisted radical prostatectomy (RARP) and external beam radiation therapy are widely regarded as gold-standard treatments for localized prostate cancer (PCa)) (1). In recent years, however, several novel and minimally invasive treatment strategies have emerged, with focal therapy (FT) gaining increasing attention as a promising alternative. FT modalities, including cryoablation, irreversible electroporation (IRE), and high-intensity focused ultrasound (HIFU), aim to selectively target tumor-bearing regions of the prostate while sparing surrounding healthy tissue (2, 3). By preserving key anatomical structures such as the neurovascular bundles, external sphincter, and urethra, FT seeks to minimize treatment-related morbidity and maintain functional outcomes, particularly urinary continence and erectile function, which are frequently compromised following RARP (4–6).

Despite these advantages, FT is associated with a clinically significant recurrence rate, with up to 20–30% of patients experiencing disease recurrence either within (“in-field”) or outside (“out-of-field”) the treated area (7–9). To date, no consensus has been reached on the optimal salvage strategy in such cases. Among the available options, salvage RARP (sRARP) represents a potentially curative approach; however, concerns remain about the impact of prior FT, particularly the thermal effects of HIFU, on the feasibility and safety of subsequent surgery.

A further challenge in interpreting existing evidence is the marked heterogeneity of patient cohorts included in prior sRARP studies. Most series have grouped together patients treated with a wide spectrum of FT modalities and extents, ranging from targeted focal ablations to more extensive hemi-gland, subtotal, or even whole-gland approaches. This variability severely limits the generalizability of published outcomes and complicates clinical decision-making (10).

To address this gap, the present single-center study aims to evaluate the feasibility, safety, oncological and functional outcomes of sRARP in a highly selected cohort of patients previously treated with targeted HIFU.

## 2 Materials and methods

### 2.1 Study design

After the approval of the local Ethics Committee of Humanitas Research Hospital (ICH-018), we performed an observational, retrospective, single-arm, single-center study based on the electronic medical record review of consecutive patients using a prospectively maintained database.

### 2.2 Patient recruitment

In this retrospective study, we evaluated patients with localized low or intermediate risk PCa, who initially underwent targeted FT as primary treatment and later required sRARP due to its failure at Humanitas Research Hospital, between June 2022 and June 2025. For the purpose of this study, targeted FT was defined as a lesion-based treatment strategy targeting the identified PCa in less than one quadrant, including a safety margin (11).

Eligible participants for HIFU treatment were adult patients (≥18 years) with clinical stage T1c or T2a, a total Prostate-Specific Antigen (PSA) ≤ 20 ng/ml, and a PSA density ≤ 0.20 ng/ml/cc. A life expectancy of ≥10 years was required. The index lesion had to be identified by at least one imaging modality among multiparametric MRI (mpMRI), microultrasound (microUS), or Prostate-Specific Membrane Antigen Positron Emission Tomography/Computed Tomography (PSMA-PET/CT). The PCa focus was defined as the lesion with the highest Society of Urological Pathology Grade Group (ISUP GG), or, when multiple foci shared the same grade, the largest focus. Lesions with a volume up to 1.5 cc were considered suitable for treatment. Pathological criteria included a histological diagnosis of conventional acinar adenocarcinoma with ISUP GG ≥ 2 confined to one lobe, a maximum grade group of 3, and cancer involvement in no more than 33% of total biopsy cores.

Exclusion criteria encompassed contraindications to HIFU treatment (very apical and anterior lesions), active perineal or pelvic inflammatory diseases, and significant cardiovascular, renal, hepatic, or respiratory conditions that could contraindicate the procedure or anesthesia, as well as active, untreated urinary tract infections.

Patients began a structured follow-up protocol starting 3 months after treatment. Visits included PSA blood tests, digital rectal examination (DRE), microUS, and standardized questionnaires covering quality of life (EQ-5D-5L), urinary function (EPIC-26 short form and IPSS), sexual function (IIEF-5), and anxiety (STAI-Y2)7–11 (12–16). At 6 months, only PSA tests and physical examinations were performed, while between 6 and 12 months, mpMRI and PSMA-PET/CT scans were added (17, 18). The 12-month assessment included PSA testing, DRE, microUS, questionnaires, and both systematic and targeted prostate biopsies when indicated. All biopsies were performed via the transperineal approach, consistent with current EAU guideline recommendations (1). Follow-up continued at 18, 24, 30, 36, 42, and 48 months, with evaluations adapted to each time point (**Supplementary Table S1**).

Among these patients, HIFU failure was defined as the occurrence of radical treatment or systemic therapy for PCa, the identification of *de novo* PCa metastases, or prostate cancer-specific death during the study period.

All focal treatments in this cohort were delivered exclusively using the Focal One^®^ semi-robotic HIFU platform (EDAP TMS, Vaulx-en-Velin, France) (19). This system integrates real-time transrectal ultrasound with mpMRI and PSMA-PET datasets through elastic fusion to generate a three-dimensional prostate model and to plan a targeted ablation zone with predefined safety margins. Energy delivery is monitored continuously and can be adjusted intraoperatively by the surgeon (19). The “double-tap” technique (repeated overlapping sonications of the same target area) was not used in this cohort.

sRARP was performed in cases of ISUP upgrading at biopsy, an increase in the number of positive cores, bilateral disease, the appearance of very apical and anterior lesions, which represent contraindications to HIFU, or patient preference.

sRARP procedures were performed by two experienced robotic surgeons (N.M.B. and G.L.) using the four-arm Da Vinci Surgical System Xi (Intuitive Surgical Inc., Sunnyvale, CA), through a transperitoneal approach according to the principles of the Montsouris technique. These included dissection of the vasa deferentia and seminal vesicles, development of the posterior plane between the prostate and rectum, bladder detachment, incision of the endopelvic fascia, suture of the dorsal venous complex, bladder neck dissection, lateral dissection of the prostate, apical dissection, posterior reconstruction, and vesicourethral anastomosis (20, 21). To determine which patients would benefit from pelvic lymphadenectomy (including internal and external iliac and obturator fossa), the 2019 Briganti nomogram or PSMA PET/CT-based lymph node uptake was used (22–24). The decision to perform nerve-sparing surgery (extrafascial, interfascial, or intrafascial) was made at the surgeon’s discretion, in accordance with European Association of Urology guidelines, in agreement with the patient and considering disease-related factors, such as the presence of extracapsular extension. Intraoperative frozen section (IFS) analysis was conducted on all patients, as per standard practice at our institution for treatment-naïve men undergoing primary RARP.

Before surgery, metastatic disease was excluded in all patients using mpMRI and PSMA PET/CT. All patients had a life expectancy of at least 10 years (25).

### 2.3 Data collection

We retrospectively collected data from patient charts, including baseline demographic and clinical characteristics such as age, sex, body mass index (BMI), comorbidities, family history of PCa, and previous prostate surgeries. Clinical and laboratory data at the time of presentation were also recorded, including PSA levels, PSA density, prostate volume, DRE findings, imaging results, and prostate biopsy outcomes. Additionally, treatment-related data were collected, including the side of treatment and the volume treated.

### 2.4 Outcome measures

The primary endpoint of this study was to assess the feasibility and safety of sRARP following targeted HIFU treatment. This was evaluated based on the rate of conversion to open surgery, operative time, feasibility of nerve-sparing and lymphadenectomy, positive surgical margin rates at both frozen section and final pathology, and the incidence of intraoperative complications. Secondary endpoints included functional outcomes, specifically urinary continence and erectile function, oncological outcomes and the incidence of early (<30 days) and late (>30 days) postoperative complications, categorized according to the Clavien-Dindo (CD) classification system (26). Postoperative biochemical response was assessed by measuring PSA levels at the first follow-up visit, scheduled 45 days after RARP, and at 1-year follow-up. Biochemical recurrence after sRARP was defined, in accordance with EAU guidelines, as a PSA ≥0.2 ng/mL confirmed by a second consecutive rising value, after an initially undetectable postoperative PSA (<0.1 ng/mL in our series). Patients whose PSA remained ≥0.1 ng/mL at the first postoperative assessment (45 days) were classified as having persistent PSA (1). Functional outcomes were assessed at the 1-year follow-up visit through patient self-report. Patients were classified as continent if they reported either full continence (no pad use) or social continence (use of no more than one pad per day) (27, 28). All patients underwent a structured urinary continence rehabilitation program after surgery, as routinely implemented at our institution (29). Potency was defined as the ability to achieve erections sufficient for penetration, with or without the use of phosphodiesterase type 5 inhibitors.

### 2.5 Statistical analysis

We analyzed the demographic, clinical, and laboratory characteristics with descriptive statistic techniques. We used the Shapiro–Wilk test to evaluate the distribution of the variables. Normally distributed continuous variables were reported as mean ± standard deviation and otherwise as the median and Q1–Q3. We reported categorical variables as absolute and relative frequencies. We used no imputation techniques for the missing data. Patients with missing values in critical variables were excluded from relevant analyses. We reported this study following the Strengthening the Reporting of Observational Studies in Epidemiology (STROBE) statement (30) and presented the STROBE checklist (**Supplementary Table S2**). We conducted all analyses using statistical software STATA/SE version 18 (StataCorp, College Station, TX, USA).

## 3 Results

### 3.1 Patient cohort and characteristics

Among 112 men treated with HIFU for localized PCa, 15 subsequently underwent sRARP between June 2022 and June 2025. The main characteristics of the patients included in the study are presented in **Table 1**. The median age of the cohort was 65 years (IQR: 60–69), with a median prostate volume of 43 cc (IQR: 38–60) and a median PSA level of 7.7 ng/mL (IQR: 5.8–8.8).

**Table 1:** Baseline pre radical treatment patients’ characteristics.

n patients	15
Median age (years)	65 (60–69)
Median PSA (ng/mL)	7.7 (5.8–8.8)
Median prostate volume (cc)	43 (38–60)
Median interval from HIFU to sRARP (months)	18 (16–18.5)
Urinary continence* n (%)	14 (93)
Erectile function^§^ n (%)	11 (73)

PSA, Prostate-Specific Antigen; pt, patient; ISUP GG, International Society of Urological Pathology Grade Group; HIFU, focal high-intensity focused ultrasound; sRARP, salvage robotic-assisted radical prostatectomy; n, number.

*self-reported full (0 pad/day) or social (≤ 1pad/day) urinary continence.

^§^self-reported erectile function sufficient for penetration, with or without phosphodiesterase type 5 inhibitors.

All patients underwent prostate biopsy prior to sRARP, which revealed ISUP GG1 in 5 patients (34%), ISUP GG2 in 8 (54%), and ISUP GG3 in 2 (12%). **Table 2** provides a summary of the total number of patients classified within each ISUP grade category at the three timepoints (pre-HIFU, pre-sRARP and post-sRARP). Notably, 5 patients (33%) experienced upgrading between the pre-HIFU biopsy and the follow-up biopsy that led to the indication for sRARP (**Supplementary Table S3**). Among patients with ISUP GG1 at the pre-sRARP biopsy, the indication for radical surgery was an increase in the number of positive cores.

**Table 2:** Distribution of patients according to ISUP grade at each timepoint.

ISUP Grade Group	Before HIFU	Before sRARP	Post sRARP
GG1, n (%)	8 (53)	5 (34)	0 (0)
GG2, n (%)	7 (47)	8 (54)	8 (54)
GG3, n (%)	0 (0)	2 (12)	5 (34)
GG4, n (%)	0 (0)	0 (0)	1 (6)
GG5, n (%)	0 (0)	0 (0)	1 (6)

ISUP GG, International Society of Urological Pathology Grade Group; sRARP, salvage robotic-assisted radical prostatectomy; HIFU, focal high-intensity focused ultrasound, n, number.

Preoperative MRI revealed a Prostate Imaging Reporting and Data System (PIRADS) score of 3 in one case (6%), PIRADS 4 in ten cases (67%), and PIRADS 5 in four cases (27%). Extracapsular extension was identified in two cases (13%). On PSMA PET imaging, no patient showed bone radiotracer uptake, while lymph node uptake was observed in seven patients (47%).

Regarding functional outcomes prior to sRARP, 14 out of 15 patients reported being continent, and 11 reported preserved erectile function, with or without phosphodiesterase type 5 inhibitors. According to the EQ-5D-5L and IIEF-5 questionnaires, median preoperative scores were 0.86 (IQR 0.82–0.90) and 18 (IQR 15–22), respectively.

### 3.2 Intraoperative and pathological outcomes

sRARP proved to be a feasible and safe procedure across all surgical steps, with no cases requiring conversion to open surgery. Bilateral nerve-sparing dissection was performed in 13 patients (87%), while the remaining two patients underwent a unilateral approach. In cases where IFS analysis was positive for cancer, additional resection of the affected area was planned to ensure complete oncological clearance. The median operative time was 187 minutes (IQR: 135–244). Extended pelvic lymph node dissection was performed in nine patients (60%), with lymph node metastases identified in one patient (11%) at final pathology. IFS analysis revealed positive surgical margins in three cases (20%), with R1 status confirmed on final pathology in two patients (13%). Final histopathological analysis revealed ISUP GG 2 in eight patients (54%), GG3 in five patients (34%), and GG4 and GG5 in one patient each (6%). An upgrading from the preoperative grade was observed in 12 out of 15 patients (80%), including one case with progression from ISUP GG3 to GG5 (**Supplementary Table S3**). Pathological T-stage was pT2 in nine patients (60%) and pT3 in six patients (40%). No intraoperative complications were reported (**Table 3**), including the absence of rectal injuries.

**Table 3:** Intraoperative and pathological outcomes.

Median operative time (minutes)	187 (135–244)
Estimated blood loss (ml)	150 (100-200)
Intraoperative transfusions, n (%)	0 (0)
Bilateral nerve-sparing dissection n (%)	13 (87)
Extended lymphadenectomy n (%)	9 (60)
PSM at IFS n (%)	3 (20)
PSM at final pathology n (%)	2 (13)
pT stage n (%):	
o ≤T2c	9 (60)
o T3a	5 (34)
o T3b	1 (6)
o T4	0 (0)
pN stage n (%):	
o N0	8 (89)
o N1	1 (11)
Intraoperative complications n (%)	0 (0)

pt, patient; IFS, intraoperative frozen section; PSM, positive surgical margins; ISUP GG, International Society of Urological Pathology Grade Group, n, number.

### 3.3 Postoperative outcomes

Early postoperative complications occurred in three patients (20%): one developed atrial fibrillation (Clavien-Dindo grade 1), one experienced wound dehiscence (Clavien-Dindo grade 2), and one developed a bilateral lymphocele requiring CT-guided drainage (Clavien-Dindo grade 3a). No late postoperative complications were reported. The median hospital stay was 4 days (IQR: 4–7). In cases where pelvic lymphadenectomy was performed, the drain was removed on postoperative day 2; otherwise, it was removed on day 1. All patients were discharged with a urinary catheter, which was removed seven days after surgery. No cases of acute urinary retention were observed following catheter removal.

At the 45-day postoperative PSA assessment, 11 patients (74%) had undetectable PSA levels, defined as <0.1 ng/mL, while four patients (26%) had persistent PSA with levels >0.1 ng/mL. These latter cases were reviewed in a multidisciplinary setting, and salvage radiotherapy was subsequently administered following sRARP. In one of these cases, radiotherapy was combined with androgen deprivation therapy.

One-year PSA data were available for 12 patients. Among them, 11 (92%) had undetectable PSA levels, while one patient had a PSA >0.2 ng/mL. This case does not meet the definition of biochemical recurrence but is best classified as persistent PSA with progression, as it involved one of the four patients who already had a PSA >0.1 ng/mL at the 45-day assessment and had undergone salvage radiotherapy. A follow-up PSMA PET scan revealed radiotracer uptake at the L2 vertebral level. The patient is currently being followed by the oncology and multidisciplinary team to determine the most appropriate treatment strategy. The median follow-up was 14 months (IQR 10.8–16.5; 95% CI).

Regarding functional outcomes, specifically urinary continence and erectile function, 12 men completed the one-year follow-up. Among them, 9 (75%) reported urinary continence at 12 months, compared to 93% prior to sRARP. Additionally, 6 patients (50%) reported erections sufficient for penetration, with or without the use of phosphodiesterase type 5 inhibitors, compared to 73% pre-sRARP (**Table 4**). According to the EQ-5D-5L and IIEF-5 questionnaires, median postoperative scores were 0.80 (IQR 0.76–0.85) and 14 (IQR 11–18), respectively.

**Table 4:** Post-operative outcomes.

Early postoperative complications n (%):	3 (20)
o CD grade 1	1 (6)
o CD grade 2	1 (6)
o CD grade ≥3a	1 (6)
Median hospital stay (days)	4 (4–7)
Median catheterization time (days)	7 (7–7)
45-day postoperative PSA assessment n (%):	
o Undetectable (<0.1 ng/mL)	11 (74)
o ≥0.1 ng/mL	4 (26)
One year postoperative PSA assessment n (%):	
o Undetectable (<0.1 ng/mL)	11 (92)
o 0.1<x<0.2 ng/mL	0 (0)
o ≥0.2 ng/mL	1 (8)
Urinary continence* at 1 year n (%)	9 (75)
Erectile function^§^ at 1 year n (%)	6 (50)

CD, Clavien-Dindo; pt, patient; PSA, Prostate-Specific Antigen, n, number.

*self-reported full (0 pad/day) or social (≤ 1pad/day) urinary continence.

^§^self-reported erectile function sufficient for penetration, with or without phosphodiesterase type 5 inhibitors.

## 4 Discussion

HIFU is increasingly being used as an alternative to radical treatments in highly selected patients with localized prostate cancer. In particular, when considering targeted FT, this approach may represent an intermediate option between active surveillance and whole-gland ablation. However, due to the limited availability of high-certainty data, especially in the setting of intermediate-risk disease, both whole-gland and focal ablative therapies should currently be limited to clinical trials or prospective registries (2).

One of the main concerns regarding FT is whether it may compromise the quality and feasibility of subsequent radical treatments (31–34). In this context, we demonstrated that sRARP following targeted HIFU is a feasible surgical option. All procedures were successfully completed robotically, with no conversions to open surgery and no intraoperative complications. Despite increased adhesions from prior tissue manipulation, surgical planes remained sufficiently preserved to allow for safe dissection and completion of the procedure.

Although we did not perform a direct comparison between sRARP and primary RARP (pRARP), nor between sRARP after focal versus whole-gland ablation, existing literature provides useful benchmarks. While sRARP can be technically more demanding, previous studies have reported similar operative times, blood loss, and complication rates compared to pRARP (33, 35). Our findings are consistent with these reports, with a high rate of bilateral nerve-sparing (87%) and a low positive surgical margin rate (13%). Among the nine patients who underwent lymphadenectomy, only one (11%) had nodal involvement. Although seven patients had PSMA-avid lymph nodes on preoperative imaging, only this single case corresponded to pathological nodal metastasis. This discrepancy may be explained by mild or equivocal PSMA uptake that does not necessarily represent metastatic disease, as well as the possibility of benign or inflammatory tracer accumulation after focal therapy. These findings reinforce that, although PSMA PET is highly sensitive, in this setting its specificity for nodal metastases remains imperfect.

Importantly, all patients in our cohort had undergone targeted HIFU, which likely contributed to the favorable surgical and functional outcomes observed. Previous studies comparing sRARP after focal versus whole-gland ablation have reported better functional outcomes in the focal group. For example, continence rates tend to be higher (69% *vs*. 54.6%) and recovery of erectile function, although overall limited, is more frequent (8.9% *vs*. 5.1%) (36). Nathan et al. also reported significantly higher pad-free continence rates in the focal group (89.3% *vs*. 53.1%), with erectile function rates of 7% and 2%, respectively (37). In our cohort, functional outcomes were even more favorable: at one year, 75% of patients were continent (either fully or socially), and 50% had preserved erectile function, with or without the use of a phosphodiesterase type 5 inhibitor. These results likely reflect both careful patient selection and the high rate of nerve-sparing procedures. Moreover, the favorable outcomes observed may also be attributed to the rigorous follow-up protocol adopted in our center for patients undergoing focal HIFU, which includes serial laboratory testing, imaging, and scheduled confirmatory biopsies. This strict monitoring allows for early identification of treatment failure, enabling timely referral for salvage surgery under optimal clinical conditions.

Regarding postoperative complications, previous reports suggest higher rates in the whole-gland ablation group (22% *vs*. 8%) (37). In our study, early postoperative complications occurred in 20% of cases; however, only one event was classified as Clavien-Dindo grade ≥3, a bilateral lymphocele requiring CT-guided drainage. No other major complications were observed.

The main limitations of this study include its observational, retrospective, and single-center design, as well as the relatively small sample size, which limited the possibility of performing propensity score matching, subgroup analyses, or multivariable regression. In addition, we did not include a matched cohort of patients undergoing primary RARP, which would have strengthened the comparative interpretation of our perioperative, functional, and oncological outcomes. The short follow-up period, particularly regarding oncological outcomes, precludes a comprehensive evaluation of long-term cancer control. Nonetheless, the primary aim of the study was to assess the feasibility of sRARP after HIFU failure, with a specific focus on early complications and functional outcomes rather than long-term oncological efficacy. In particular, our favorable outcomes derive from a small and highly selected cohort of patients treated with targeted HIFU, and therefore may not be generalizable to patients undergoing sRARP after more extensive HIFU ablation. A major strength of this study lies in the homogeneous selection of patients who had previously undergone targeted HIFU, offering a unique and well-defined clinical setting for the evaluation of the outcomes of interest. Further studies with longer follow-up and larger cohorts are warranted to better characterize long-term outcomes.

## 5 Conclusion

This study is one of the few single-center analyses investigating RARP as a salvage option in a carefully selected cohort of patients previously treated with targeted HIFU. Our findings suggest that targeted HIFU does not compromise the feasibility or safety of subsequent radical surgery. This is supported by operative times, complication rates and the favorable functional and oncological outcomes observed in our series, despite the intrinsic limitations of a retrospective small-cohort design. Moreover, the structured and rigorous follow-up protocol adopted at our center likely contributed to the early identification of HIFU failure and timely referral for salvage surgery under optimal conditions.

## Data Availability

The raw data supporting the conclusions of this article will be made available by the authors, without undue reservation.
