# Supplementary material for: Salvage robotic-assisted radical prostatectomy after targeted high-intensity focused ultrasound: a single-center study on feasibility, oncological and functional outcomes
Source: Front Oncol. 2026 Feb 6;16:1711853. doi: 10.3389/fonc.2026.1711853 (PMC12920220; doi:10.3389/fonc.2026.1711853)
Supplement: Supplementary file 1 [file DataSheet1.docx]

Supplementary Material

**eTable 1 - Follow-up protocol for patients undergoing high-intensity focused ultrasound**

PSA was performed at 0, 3, 6, 12, 18, 24, 30, 36, 42, and 48 months. DRE was performed at 0, 3, 6, 12, 18, 24, 36, and 48 months. Questionnaires and Micro-US collected at 0, 3, 12, 24, 36, and 48 months. mpMRI was performed at baseline (0 months), between 6 and 9 months, and at 24, 36, and 48 months. PET-PUMA was performed at baseline, between 6 and 9 months, and at 24 and 48 months. Prostate biopsy was performed at baseline, at 12 months, and at 48 months.


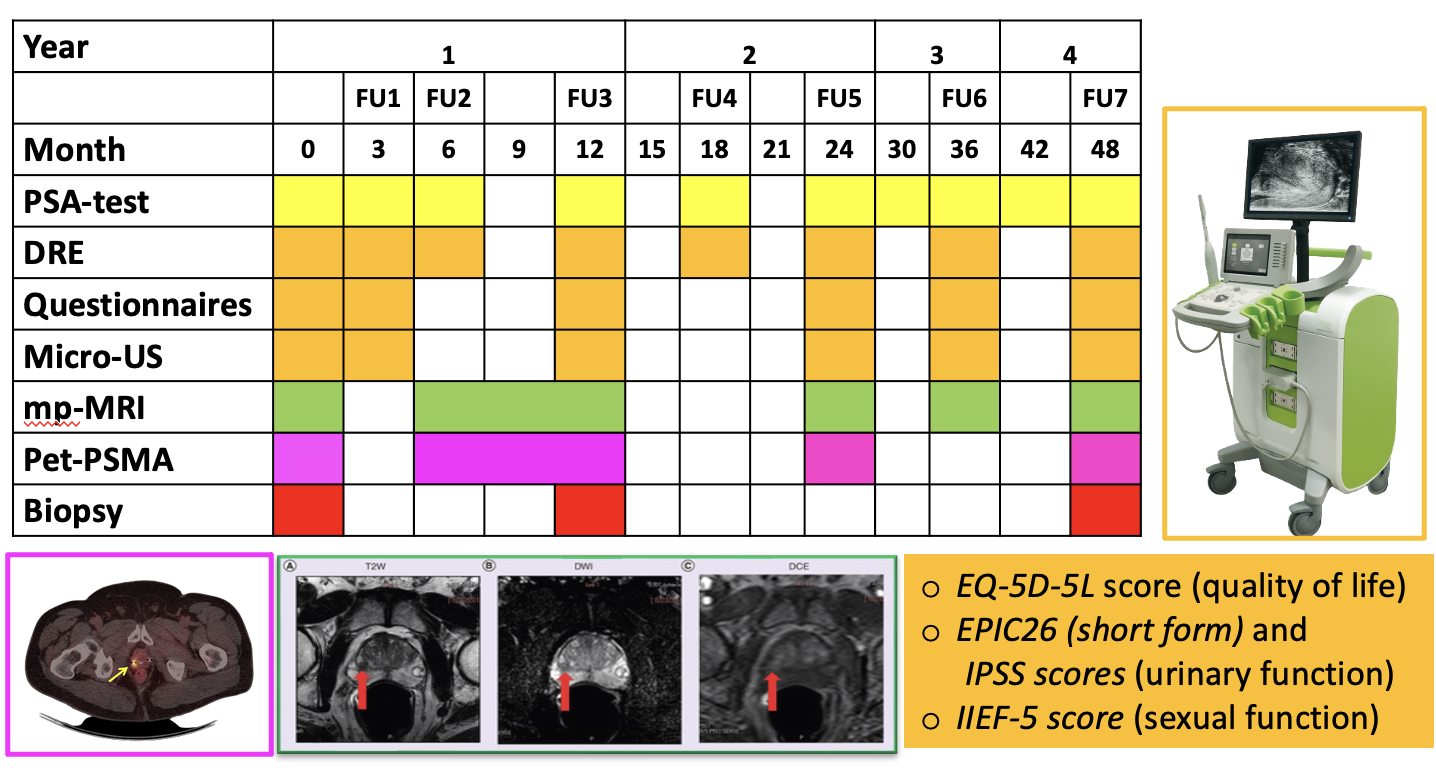


*FU: follow-up ; DRE : Digital Rectal Examination ; PSA: Prostate-Specific Antigen; Micro-US: High-resolution Micro-Ultrasound; mp-MRI: Multiparametric Magnetic Resonance Imaging; Pet-PSMA: Prostate-Specific Membrane Antigen Positron Emission Tomography*

**eTable 2 - STROBE Checklist for Observational Studies**

| Item | Recommendation | Page No. | Relevant text from manuscript |
| --- | --- | --- | --- |
| 1 | Title and abstract | 1 | Title: 'Salvage Robotic-Assisted Radical Prostatectomy After Truly Focal High-Intensity Focused Ultrasound: A Single-Center Study on Feasibility, Oncological and Functional Outcomes'. Abstract includes study design, aim, methods, results, and conclusions. |
| 2 | Background/rationale | 2 | ‘FT modalities, including cryoablation, irreversible electroporation (IRE), and high-intensity focused ultrasound (HIFU), aim to selectively target tumor-bearing regions of the prostate while sparing surrounding healthy tissue.’ |
| 3 | Objectives | 2 | ‘This study aimed to evaluate feasibility, functional, and early oncological outcomes of sRARP in this specific setting.’ |
| 4 | Study design | 3 | ‘We performed an observational, retrospective, single-arm, single-center study based on the electronic medical record review of consecutive patients using a prospectively maintained database.’ |
| 5 | Setting | 3 | ‘Between June 2022 and June 2025 at Humanitas Research Hospital…’ |
| 6a | Participants | 3–4 | Eligibility criteria for HIFU and sRARP are provided in detail under 'Patient Recruitment'. Includes clinical stage, PSA thresholds, ISUP grade, lesion volume, and exclusion criteria. |
| 7 | Variables | 4–5 | ‘We retrospectively collected data from patient charts, including baseline demographic and clinical characteristics such as age, sex, BMI, comorbidities...’ |
| 8 | Data sources/measurement | 4 | ‘We retrospectively collected data from patient charts…’ and described outcome measures in section 2.4. |
| 9 | Bias | 9 | ‘The main limitations of this study include its observational, retrospective, and single-center design, as well as the relatively small sample size…’ |
| 10 | Study size | 6 | ‘Among 112 men treated with HIFU for localized PCa, 15 subsequently underwent sRARP between June 2022 and June 2025.’ |
| 11 | Quantitative variables | 5 | ‘Continence outcomes were assessed at the 1-year follow-up, with success defined as either complete continence (no pad use) or social continence (use of no more than one pad per day).’ |
| 12a | Statistical methods | 5 | ‘We analyzed the demographic, clinical, and laboratory characteristics with descriptive statistic techniques…’ |
| 14a | Descriptive data | 6 | ‘The main characteristics of the patients included in the study are presented in Table 1.’ |

**eTable 3 - Patient-level changes in ISUP grade over time**

***Color code***: Red: worsening of the ISUP GG compared to the previous assessment. Gray: ISUP GG remained unchanged compared to the previous assessment.

|  | **ISUP GG before HIFU** | **ISUP GG before sRARP** | **ISUP GG post sRARP** |
| --- | --- | --- | --- |
| Patient 1 | 1 | 1 | 2 |
| Patient 2 | 1 | 2 | 3 |
| Patient 3 | 1 | 1 | 2 |
| Patient 4 | 2 | 2 | 3 |
| Patient 5 | 2 | 2 | 3 |
| Patient 6 | 1 | 1 | 2 |
| Patient 7 | 2 | 2 | 2 |
| Patient 8 | 2 | 3 | 5 |
| Patient 9 | 1 | 2 | 2 |
| Patient 10 | 1 | 2 | 2 |
| Patient 11 | 1 | 1 | 2 |
| Patient 12 | 1 | 1 | 2 |
| Patient 13 | 2 | 2 | 3 |
| Patient 14 | 2 | 3 | 4 |
| Patient 15 | 2 | 2 | 3 |

*ISUP GG: International Society of Urological Pathology Grade Group; sRARP: salvage robotic-assisted radical prostatectomy; HIFU: focal high-intensity focused ultrasound.*
